# Definitions of clinical study outcome measures for cardiovascular diseases: the European Unified Registries for Heart Care Evaluation and Randomized Trials (EuroHeart)

**DOI:** 10.1093/eurheartj/ehae724

**Published:** 2024-11-15

**Authors:** Chris Wilkinson, Asad Bhatty, Gorav Batra, Suleman Aktaa, Adam B Smith, Jeremy Dwight, Marcin Ruciński, Sam Chappell, Joakim Alfredsson, David Erlinge, Jorge Ferreira, Ingibjörg J Guðmundsdóttir, Þórdís Jóna Hrafnkelsdóttir, Inga Jóna Ingimarsdóttir, Alar Irs, András Jánosi, Zoltán Járai, Manuel Oliveira-Santos, Bogdan A Popescu, Peter Vasko, Dragos Vinereanu, Jonathan Yap, Raffaele Bugiardini, Edina Cenko, Ramesh Nadarajah, Matthew R Sydes, Stefan James, Aldo P Maggioni, Lars Wallentin, Barbara Casadei, Chris P Gale, Global Cardiovascular Outcomes Consortium and in collaboration with ACNAP, ACVC, EACVI, EAPC, EAPCI, EHRA, ESC Committee for Young CV Professionals, ESC Registry Committee, HFA, ESC Patient Forum and these Working Groups: aorta and peripheral vascular diseases, atherosclerosis and vascular biology, cardiac cellular electrophysiology, cardiovascular pharmacotherapy, cardiovascular regenerative and restorative medicine, cardiovascular surgery, cellular biology of the heart, e-cardiology, myocardial function, pulmonary circulation and right ventricular function and thrombosis, Victor Aboyans, Ana G Almeida, Dan Atar, Gorav Batra, Antoni Bayés-Genís, Asad Bhatty, Giuseppe Biondi-Zoccai, Marc P Bonaca, Nikolaos Bonaros, Bianca J J M Brundel, Raffaele Bugiardini, Gianluca Campo, Barbara Casadei, Ruben Casado-Arroyo, Claudio Ceconi, Edina Cenko, Ovidiu Chioncel, Michele Ciccarelli, Louise Coats, Jean-Philippe Collet, Gheorghe-Andrei Dan, Victoria Delgado, Polychronis Dilaveris, Dobromir Dobrev, Erwan Donal, David Duncker, Sarah Moharem Elgamal, Justin A Ezekowitz, Gregg Fonarow, Alan G Fraser, Chris P Gale, George Giannakoulas, Bruna Gigante, Massimiliano Gnecchi, Can Gollmann-Tepeköylü, Stephen J Greene, Jordi Heijman, Jonathan Howes, Bernard Iung, Stefan James, Magnus T Jensen, Vijay Kunadian, Malgorzata Lelonek, Sergio Leonardi, Erik Lerkevang Grove, Luca Liberale, Riccardo Liga, A Michael Lincoff, Roberto Lorusso, Aldo P Maggioni, Mamas Mamas, Olivia Manfrini, Fabio Mangiacapra, Nina Ajmone Marsan, María Martín-Fernandez, Jose L Merino, Marco Metra, Alessandro Parolari, Cinzia Perrino, Lorenz Räber, Benyamin Rahmani, Peter P Rainer, Giuseppe M C Rosano, Alexia Rossi, Andrea Rubboli, Tanja Rudolph, Sigrid Sandner, Gianluigi Savarese, Jolanta Siller-Matula, Samuel Sossalla, Cristiano Spadaccio, Eugenio Stabile, David Tanne, Jurrien ten Berg, Matthias Thielmann, Roderick W Treskes, Izabella Uchmanowicz, Jacob A Udell, Roland R J van Kimmenade, Lars Wallentin, Chris Wilkinson, Marija Zdravkovic

**Affiliations:** Hull York Medical School, University of York, YO10 5DD York, UK; Academic Cardiovascular Unit, South Tees NHS Foundation Trust, James Cook University Hospital, Middlesbrough, UK; Leeds Institute of Cardiovascular and Metabolic Medicine, University of Leeds, Leeds, UK; Leeds Institute for Data Analytics, University of Leeds, Leeds, UK; Department of Cardiology, Leeds Teaching Hospitals NHS Trust, Leeds, UK; Department of Medical Sciences, Cardiology and Uppsala Clinical Research Centre, Uppsala University, Uppsala, Sweden; Leeds Institute of Cardiovascular and Metabolic Medicine, University of Leeds, Leeds, UK; Leeds Institute for Data Analytics, University of Leeds, Leeds, UK; Department of Cardiology, Leeds Teaching Hospitals NHS Trust, Leeds, UK; Leeds Institute of Cardiovascular and Metabolic Medicine, University of Leeds, Leeds, UK; Leeds Institute for Data Analytics, University of Leeds, Leeds, UK; European Society of Cardiology Patient Forum; European Society of Cardiology Patient Forum; Leeds Institute of Cardiovascular and Metabolic Medicine, University of Leeds, Leeds, UK; Leeds Institute for Data Analytics, University of Leeds, Leeds, UK; Department of Cardiology, Linköping University Hospital, Linköping, Sweden; Department of Clinical Sciences, Lund University, Lund, Sweden; Department of Cardiology, Hospital de Santa Cruz, Centro Hospitalar de Lisboa Ocidental, Carnaxide, Portugal; Department of Cardiology, Landspitali University Hospital, Reykjavik, Iceland; Department of Cardiology, Landspitali University Hospital, Reykjavik, Iceland; Department of Cardiology, Landspitali University Hospital, Reykjavik, Iceland; Department of Health Sciences, Faculty of Medicine, University of Iceland, Reykjavik, Iceland; Heart Clinic, Tartu University Hospital, Tartu, Estonia; György Gottsegen National Cardiovascular Institute, Budapest, Hungary; Department of Cardiology, South Buda Center Hospital, Szent Imre Teaching Hospital, Budapest, Hungary; Cardiology Department, Unidade Local de Saúde de Coimbra, Coimbra, Portugal; Cardiology Clinic, University of Medicine and Pharmacy Carol Davila, Emergency Institute for Cardiovascular Diseases Prof Dr C C Iliescu, Bucharest, Romania; Department of Cardiology, Linköping University Hospital, Linköping, Sweden; Cardiology Department, Unidade Local de Saúde de Coimbra, Coimbra, Portugal; Cardiology and Cardiovascular Surgery, University and Emergency Hospital, Bucharest, Romania; Department of Cardiology, National Heart Centre Singapore, Singapore; Department of Experimental, Diagnostic and Specialty Medicine, Department of Medical and Surgical Sciences, University of Bologna, Bologna, Italy; Department of Experimental, Diagnostic and Specialty Medicine, Department of Medical and Surgical Sciences, University of Bologna, Bologna, Italy; Leeds Institute of Cardiovascular and Metabolic Medicine, University of Leeds, Leeds, UK; Leeds Institute for Data Analytics, University of Leeds, Leeds, UK; Department of Cardiology, Leeds Teaching Hospitals NHS Trust, Leeds, UK; BHF Data Science Centre, HDR UK, London, UK; MRC Clinical Trials Unit at UCL, Institute of Clinical Trials and Methodology, UCL, London, UK; Department of Medical Sciences, Cardiology and Uppsala Clinical Research Centre, Uppsala University, Uppsala, Sweden; ANMCO Research Centre, Heart Care Foundation, 50121 Florence, Italy; Department of Medical Sciences, Cardiology and Uppsala Clinical Research Centre, Uppsala University, Uppsala, Sweden; Division of Cardiovascular Medicine, NIHR Oxford Biomedical Research Centre, University of Oxford, Oxford, UK; Leeds Institute of Cardiovascular and Metabolic Medicine, University of Leeds, Leeds, UK; Leeds Institute for Data Analytics, University of Leeds, Leeds, UK; Department of Cardiology, Leeds Teaching Hospitals NHS Trust, Leeds, UK

**Keywords:** Acute coronary syndrome, Atrial fibrillation, Heart failure, Transcatheter aortic valve intervention, Outcomes, Registry, data, EuroHeart

## Abstract

**Background and Aims:**

Standardized definitions for outcome measures in randomized clinical trials and observational studies are essential for robust and valid evaluation of medical products, interventions, care, and outcomes. The European Unified Registries for Heart Care Evaluation and Randomised Trials (EuroHeart) project of the European Society of Cardiology aimed to create international data standards for cardiovascular clinical study outcome measures.

**Methods:**

The EuroHeart methods for data standard development were used. From a Global Cardiovascular Outcomes Consortium of 82 experts, five Working Groups were formed to identify and define key outcome measures for: cardiovascular disease (generic outcomes), acute coronary syndrome and percutaneous coronary intervention (ACS/PCI), atrial fibrillation (AF), heart failure (HF) and transcatheter aortic valve implantation (TAVI). A systematic review of the literature informed a modified Delphi method to reach consensus on a final set of variables. For each variable, the Working Group provided a definition and categorized the variable as mandatory (Level 1) or optional (Level 2) based on its clinical importance and feasibility.

**Results:**

Across the five domains, 24 Level 1 (generic: 5, ACS/PCI: 8, AF: 2; HF: 5, TAVI: 4) and 48 Level 2 (generic: 18, ACS-PCI: 7, AF: 6, HF: 2, TAVI: 15) outcome measures were defined.

**Conclusions:**

Internationally derived and endorsed definitions for outcome measures for a range of common cardiovascular diseases and interventions are presented. These may be used for data alignment to enable high-quality observational and randomized clinical research, audit, and quality improvement for patient benefit.

## Structured Graphical Abstract

**Structured Graphical Abstract:**
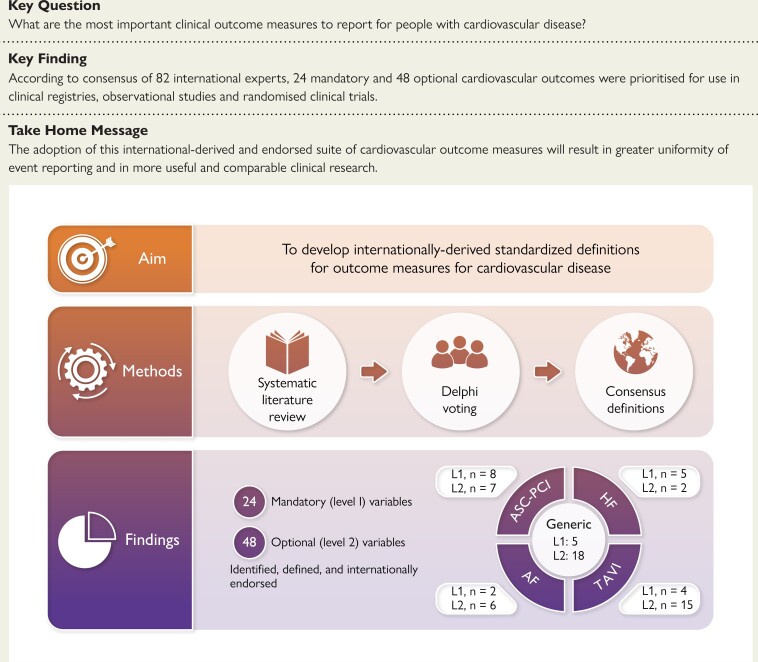
Internationally derived, standardized definitions for outcome measures in people with cardiovascular disease. ACS-PCI, acute coronary syndrome-percutaneous coronary intervention; HF, heart failure; AF, atrial fibrillation; TAVI, transcatheter aortic valve intervention

**See the editorial comment for this article ‘Data science from EuroHeart: a job in hand’, by A. Timmis, https://doi.org/10.1093/eurheartj/ehae819.**

## Introduction

Careful selection and use of clinical outcome measures is of paramount importance to enable valid and reliable quantification of the benefits and harms of different management strategies.^1,2^ Sets of outcome measures have been proposed for cardiovascular disease but these are often limited to single conditions or diseases,^3–5^ or are specific to individual randomized clinical trials (RCT),^6^ and lack a structured hierarchy.^3–6^ At time of development, existing outcome sets have often not included patient stakeholder involvement.^7^

The definitions of cardiovascular outcomes measures employed in observational research have been inconsistent and heterogeneous,^8^ yet an increasing proportion of RCTs are using routinely collected healthcare systems and/or clinical registry data for outcome evaluation.^9–12^ Registry-based RCTs now span multiple geographies.^9^ It is therefore increasingly important that consistent, internationally endorsed and robustly defined clinical outcome measures are established.^13^

The European Unified Registries for Heart Care Evaluation and Randomised Trials (EuroHeart) project of the European Society of Cardiology (ESC) has previously published data standards for four common cardiovascular domains: acute coronary syndrome and percutaneous coronary intervention (ACS/PCI), atrial fibrillation (AF), heart failure (HF), and transcatheter aortic valve implantation (TAVI).^14–17^ These were developed using a standardized, evidence-based method,^18^ which we now use to select and define cardiovascular outcome measures for assessments in common cardiovascular diseases and interventions. This catalogue of cardiovascular outcome measures will provide a ‘common language’ to facilitate federated, pooled, comparative and meta-analyses of independent yet harmonized data sets, and support the delivery of international registry-based RCTs. It will also improve clinical event reporting—making clinical studies more robust, generalizable, and applicable—and as such it will advance evidence-based assessment of the effectiveness of cardiovascular care.

## Methods

We classified cardiovascular outcome measures as either generic or domain-specific. Generic measures were defined as those of potential applicability to all patients with cardiovascular diseases. Domain-specific variables were defined as those applying to patients following diagnosis of ACS, AF, or HF, or after a TAVI procedure. Every participant would be eligible for the collection of generic outcomes in addition to specific domains.

### EuroHeart method

We followed the EuroHeart method for cardiovascular data standard development.^18^ This involved: (i) completion of a systematic review of the literature to synthesize a list of ‘candidate’ variables; (ii) selection and prioritisation of variables by domain experts in the Working Group using a modified Delphi method; and (iii) Working Group feedback.^18^

### Systematic literature review

The protocol was pre-registered.^19^ We searched Embase and Ovid Medline for studies published in the three medical journals with the highest impact factor: New England Journal of Medicine (NEJM), Lancet or Journal of the American Medical Association (JAMA) on or after 1st January 2000 until 12th October 2021. Studies were included if they reported results from a Phase 3 RCT or a multi-centre observational study, and included adults with coronary artery disease, ACS, PCI, heart rhythm disease, cardiomyopathy, HF, or valve disease, to ensure coverage of all five domains. Conference abstracts or review articles were excluded, as were sub-studies where the main paper was included in the review, and studies in which clinical outcomes were not reported or not defined. The search strategy was developed with a research librarian (see supplementary data online, Tables*S1* and *S2*). Variables that were included in the existing domain registries were reviewed,^14–17^ and outcome measure variables were included for Delphi voting. These were presented to the Working Group participants, who were able to suggest additional variables based upon clinical expertise.

### Working groups

We approached all of the ESC Associations and Working Groups to nominate individuals with relevant clinical expertise to join a Global Cardiovascular Outcomes Consortium, alongside existing members of the EuroHeart team. Additional international clinical experts were approached directly for their specific expertise in clinical trials, registries, and regulators. From this Consortium, five Working Groups were assembled: one for the generic cardiovascular outcome measures, and one for each of the four EuroHeart domains. Some people participated in more than one group, depending on their experience and availability (see Supplementary data).

### Variable level

Working group members were asked to take part in a Delphi process to consider three options for each proposed cardiovascular outcome measure variable: include as a mandatory (Level 1) variable; include as an optional (Level 2) variable; or do not include. Voting was conducted in an online poll. Level 1 cardiovascular outcome measure variables are intended for collection in all participants in the registry, whereas Level 2 variables are discretionary and may be useful and available in some (but not all) settings and countries, depending on the purpose of the registry.

Data were collated in MS Excel for calculation of voting proportions. The threshold for inclusion as a Level 1 variable was at least 75% of participants voting for selection of the variable as Level 1.^18^ The threshold for inclusion as a Level 2 variable was at least 75% of participants selecting for the variable either Levels 1 or 2. Where a cardiovascular outcome measure variable was already included as a Level 1 variable in the generic domain, this was not considered again by the other domain groups (as it would already apply to all registry participants). Where a variable was already included as a Level 2 variable in the generic domain, it could be re-considered by the Working Group for upgrade to Level 1 within that specific domain.

### Selection of the final set of variables

The results of the Delphi voting were presented in an online meeting, and the results of each cardiovascular outcome measure variable were discussed. Where a Level 1 confirmatory vote had been made already, these results were presented for information only—because the threshold for inclusion had been made. Where the threshold for Levels 1 or 2 inclusion had not been reached, participants could request for this to be re-phrased based upon their clinical expertise, and this could proceed for a second vote with the same thresholds employed as in the first round.

### Definitions

The proposed definitions for each variable were collated from the literature and shared with the invitees from every Working Group for their comment and clarification. These were agreed by consensus. The previously published variables and definitions for each domain included a range of classification systems for bleeding;^14–17^ for this outcomes domain, however, participants were clear that a single harmonized classification for bleeding outcome measure was essential. Accordingly, an online poll was circulated to all participants to vote on their preference for the Valve Academic Research Consortium (VARC),^20^ Bleeding Academic Research Consortium (BARC),^21^ or International Society On Thrombosis and Haemostasis (ISTH)^22^ classifications of bleeding that would then be employed across all of the cardiovascular outcome domains.

### Patient and public involvement

The ESC Patient Forum was invited to contribute to this project from its inception. Their feedback was that the development of data variables and standards for cardiovascular outcomes measures was too technical for their meaningful contribution. Instead, they suggested that the results of the Delphi polls were presented to the Patient Forum for their discussion and comment, which took place in December 2023 prior to the finalisation of the catalogue of cardiovascular outcome measures. Representatives from the ESC Patient Forum are also part of the research team for the development of patient-reported outcome measures.

## Results

### Systematic review

Of 4728 publications that were screened, 801 (16.9%) were included in the review after full-text evaluation. Of these, 320 (40.0%) were published in the NEJM, 284 (35.5%) in JAMA, and 197 (24.6%) in the Lancet, comprising 620 (77.4%) RCTs and 181 (22.6%) observational studies. The most frequently reported primary outcome measure was a composite (449 studies, 56.1%), followed by all-cause mortality (109 studies, 13.6%). Where a composite was the primary outcome measure, the most frequent components were myocardial infarction (273 studies, 60.8%), all-cause mortality (242 studies, 53.9%), stroke (190 studies, 42.3%), and cardiovascular mortality (178 studies, 39.6%).

### The working group process

**Figure 1:**
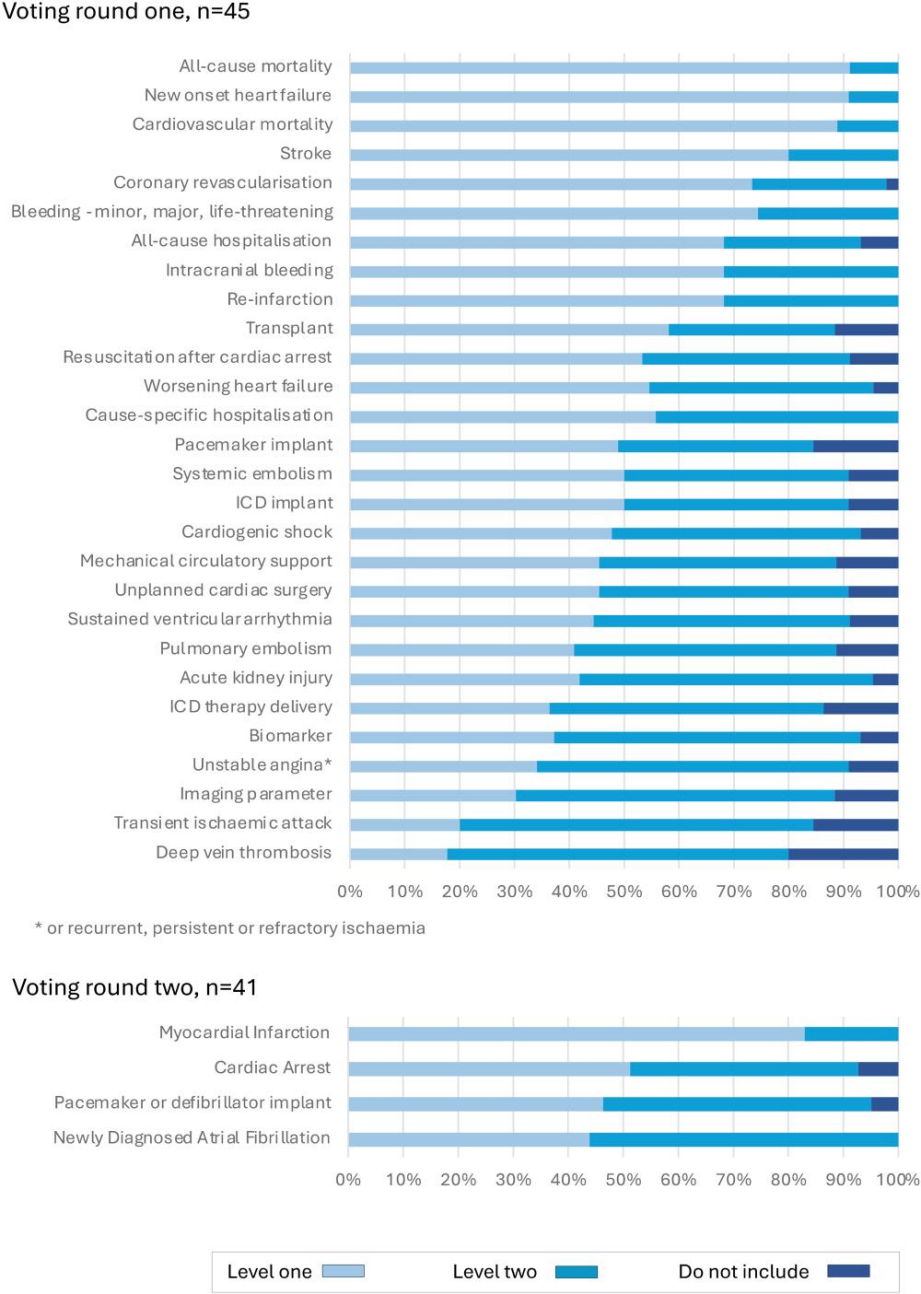
Voting results, generic domain. ICD, implantable cardioverter defibrillator. Level 1: mandatory variable; Level 2: optional variable

**Figure 2:**
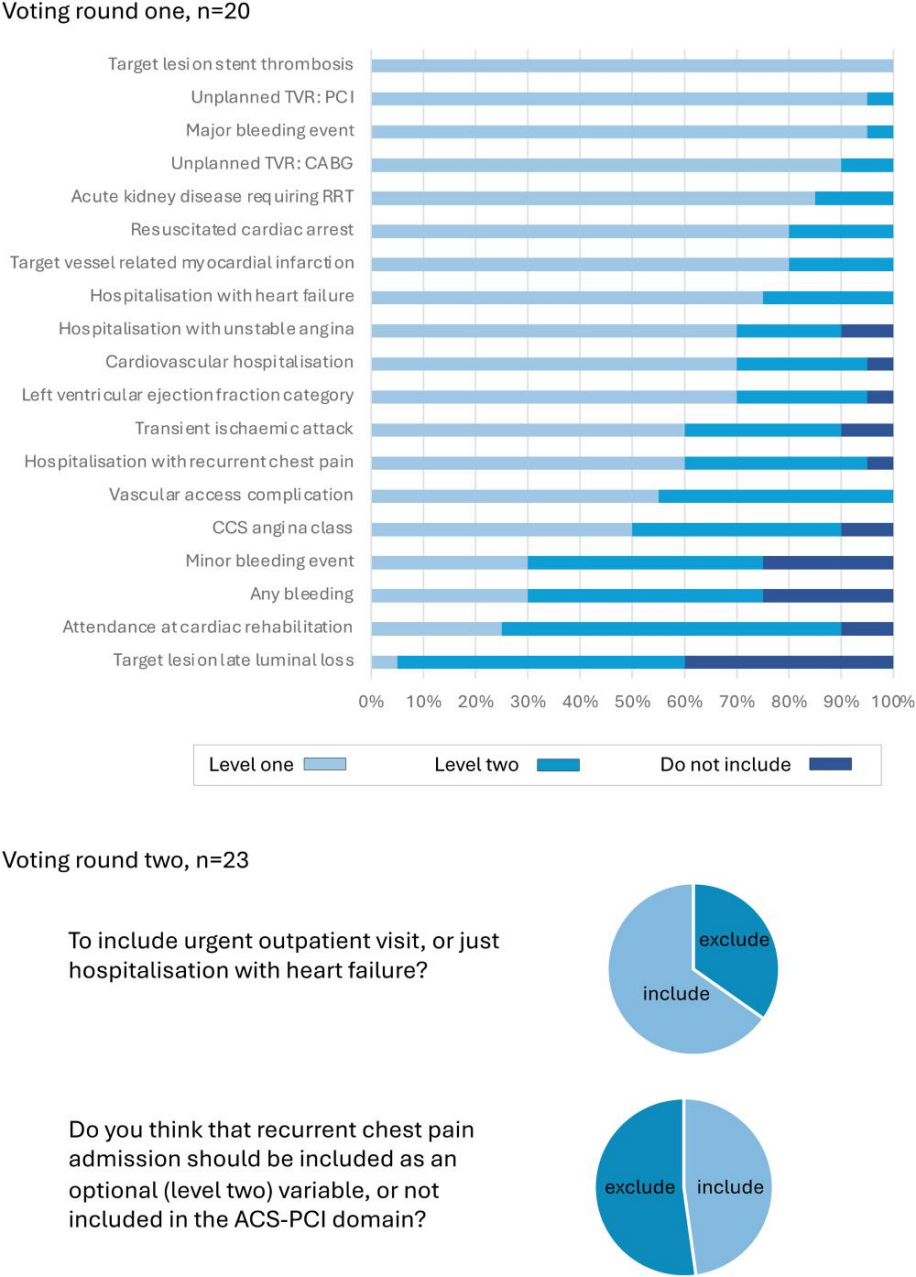
Voting results, acute coronary syndrome/percutaneous coronary intervention domain. CABG, coronary artery bypass grafting surgery; CCS, Canadian Cardiovascular Society; ICD, implantable cardioverter defibrillator; PCI, percutaneous coronary intervention; RRT, renal replacement therapy; TVR, target vessel revascularisation. Level 1: mandatory variable; Level 2: optional variable

**Figure 3:**
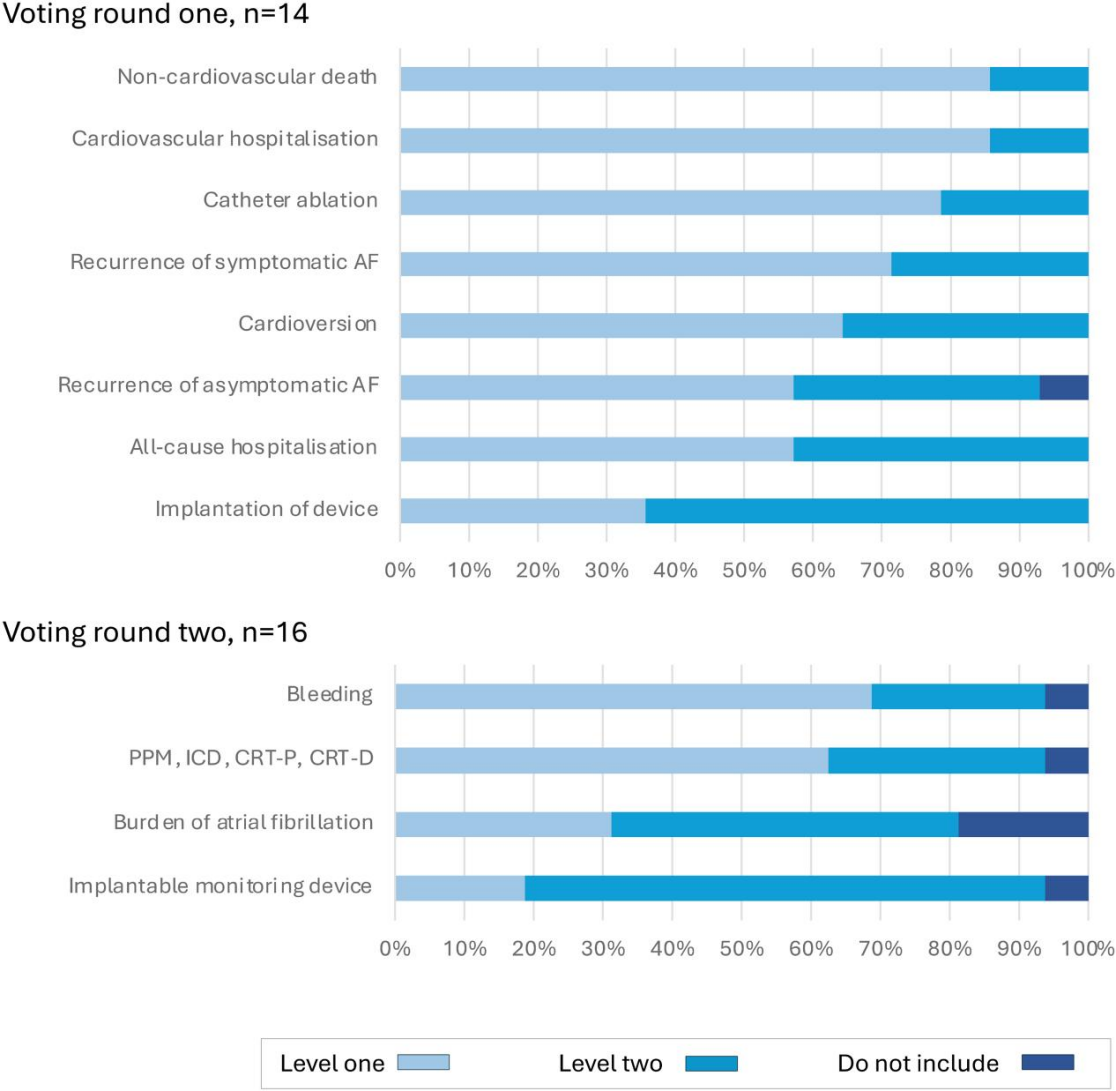
Voting results, AF domain. AF, atrial fibrillation/flutter; PPM, permeant pacemaker; ICD, implantable cardioverter defibrillator; CRT-P, cardiac resynchronisation pacemaker; CRT-D, cardiac resynchronisation defibrillator. Level 1: mandatory variable; Level 2: optional variable

**Figure 4:**
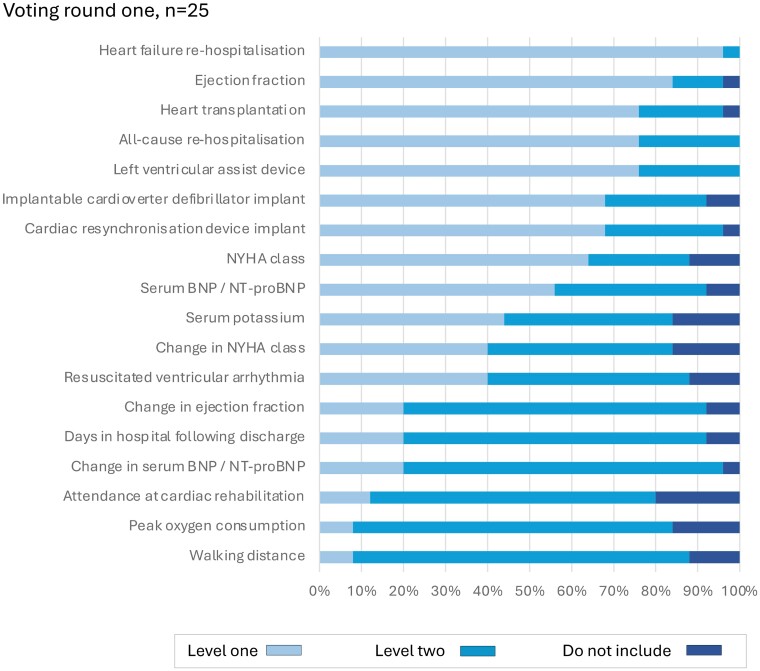
Voting results, heart failure domain. NT-proBNP/BNP, N-Terminal Pro/B-type Natriuretic Peptide; NYHA, New York Heart Association. Level 1: mandatory variable; Level 2: optional variable

**Figure 5:**
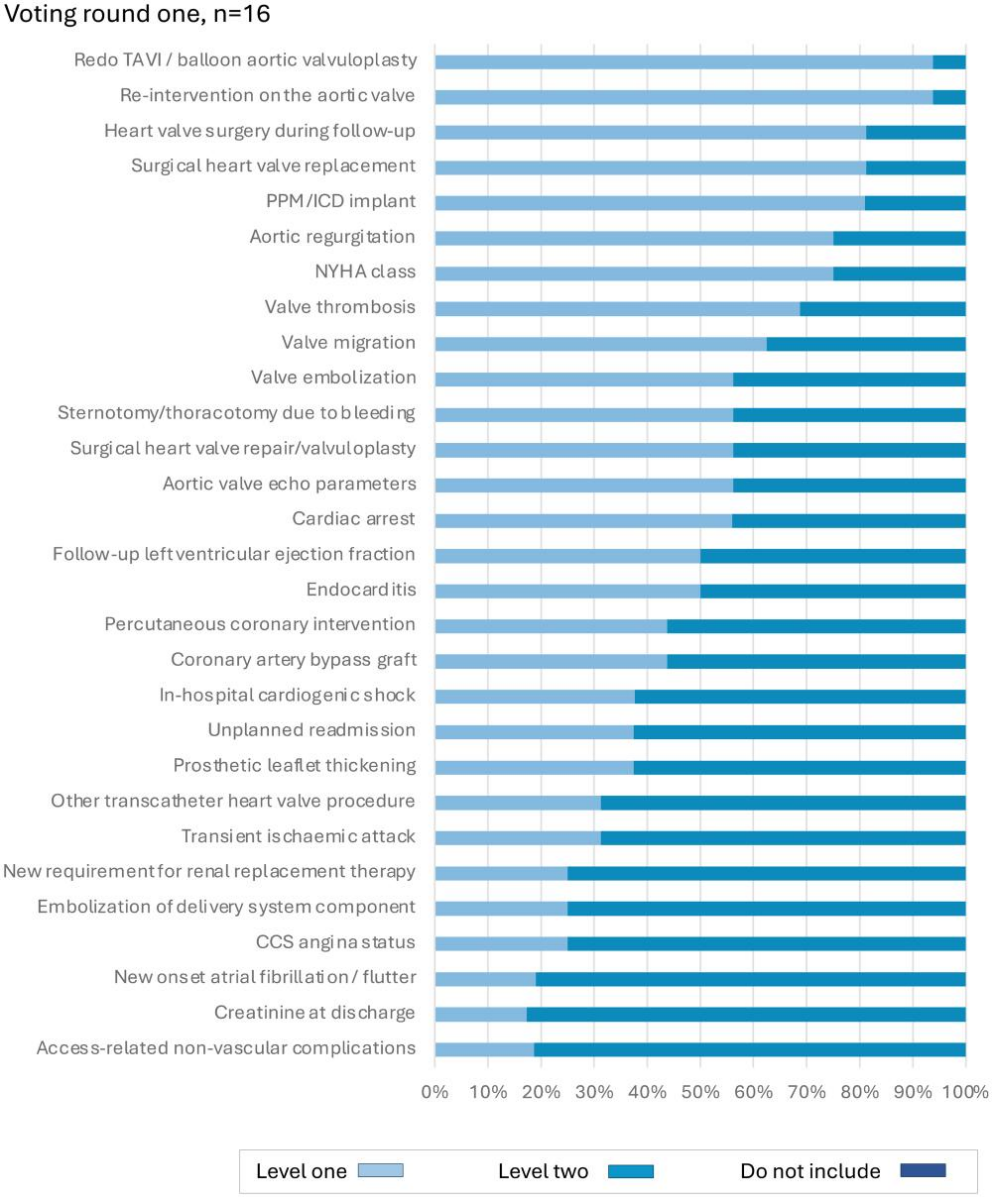
Voting results, transcatheter aortic valve intervention domain. CCS, Canadian Cardiac Society; ICD, implantable cardioverter defibrillator; NYHA, New York Heart Association; PPM, permanent pacemaker; TAVI, transcatheter aortic valve intervention. Level 1: mandatory variable; Level 2: optional variable

The extracted clinical outcome measures were presented to the five Working Groups for consideration as candidate variables for inclusion. In the first round, 28 candidate variables were considered for the generic cardiovascular outcome measures domain by 45 experts (*Figure 1*). Following discussion, a further four variables were considered by 41 experts (51 individuals contributed in total). For the ACS/PCI outcome measures domain, 26 experts reviewed 19 candidate variables in two rounds of voting (*Figure 2*). For the AF outcome measures domain, 18 experts reviewed 12 variables in two rounds of voting (*Figure 3*). For the HF outcome measures domain, 31 experts reviewed 19 variables in two rounds of voting (*Figure 4*). In the TAVI outcome measures domain, all 29 variables were already included in the TAVI registry, and so the reference group were asked to consider whether these should be considered as Levels 1 or 2 outcomes, and then the list refined during the meeting, during which 16 experts reviewed 29 variables in one round of voting (*Figure 5*). The complete list of variables and their definitions was reviewed by all contributors (*n* = 82).

### Generic domain: Level 1 variables

For the generic cardiovascular outcome measures domain, five Level 1 variables were agreed: all-cause mortality, cardiovascular mortality, myocardial infarction, stroke, and HF (*Table 1*).

**Table 1:** Generic domain—clinical outcomes and their definitions

**Generic domain: Level 1 variables**	
All-cause mortality	Death from any cause
Cardiovascular mortality	Death that is primarily from a cardiovascular cause: Related to HF, cardiogenic shock, native, mechanical or bioprosthetic valve dysfunction, myocardial infarction, stroke, thromboembolism, bleeding, tamponade, vascular complication, arrhythmia or conduction system disturbances, cardiovascular infection (e.g. mediastinitis, endocarditis), or other clear cardiovascular cause. Intraprocedural death—caused by immediate/latent complications of a cardiovascular procedure. Sudden death—Sudden natural death presumed to be of cardiac cause that occurs within 1 h of onset of symptoms in witnessed cases, and within 24 h of last being seen alive when it is unwitnessed. In autopsied cases, it is defined as the natural unexpected death of unknown or cardiac cause. Death of unknown causes.^6,20,46^
Myocardial infarction	Myocardial infarction, as defined according to the latest universal definition of MI, currently: a rise and/or fall of cardiac troponin with at least one value above the 99th percentile and/or symptoms suggestive of ischaemia, new significant ECG changes, imaging evidence of new loss of viable myocardium or new regional wall motion abnormality in a pattern consistent with an ischaemic aetiology or identification of a coronary thrombus by angiography/intracoronary imaging or by autopsy. History of myocardial infarction also includes episodes of symptoms suggestive of myocardial ischaemia, which are accompanied by presumed new ischaemic ECG changes or ventricular fibrillation; coronary intervention-related myocardial infarction; and CABG-related myocardial infarction.^47^
Stroke	An acute episode of focal or global neurological dysfunction (lasting for ≥ 24 h or until death) caused by an infarction or haemorrhage in the brain, spinal cord, or retina resulting in cell damage based on pathological, imaging, or other objective evidence. Stroke does not include non-vascular neurological deficits. Ischaemic stroke is defined as an acute episode of focal, cerebral, spinal, or retinal dysfunction that is caused by central nervous system infarction, where the neurological dysfunction lasts for ≥24 h. Ischaemic stroke may result in haemorrhage (haemorrhagic transformation). Haemorrhagic stroke is defined as an acute episode of focal or global neurological dysfunction of the brain, spinal cord or retina that is caused by a spontaneous (not traumatic) collection of intraparenchymal, intraventricular, and/or sub-arachnoid blood, where the neurological dysfunction lasts for ≥24 h. Haemorrhagic stroke does not include sub-dural hematomas. Unspecified stroke is defined as an acute episode of focal or global neurological dysfunction that is caused by a presumed infarction or haemorrhage to the central nervous system, where the neurological dysfunction lasts for ≥24 h but with insufficient information to allow categorisation as either ischaemic or haemorrhagic stroke.^48^
HF	A new clinical diagnosis of HF made by a healthcare professional. HF is a clinical syndrome characterized by typical symptoms (e.g. dyspnoea) and/or signs (e.g. ankle swelling), caused by a structural and/or functional cardiac abnormality (e.g. left ventricular hypertrophy or impairment), and associated with elevated natriuretic peptide levels and/or objective evidence of pulmonary or systemic congestion from a cardiogenic origin at rest or with exercise.^49^
**Generic domain: Level 2 variables**	
Acute kidney injury	Increase in serum creatinine by ≥0.3 mg/dL (≥26.5 µmol/L) within 48 h; or an increase in serum creatinine to ≥1.5 times baseline, which is known or presumed to have occurred within the prior 7 days; or urine volume <0.5 mL/kg/h for 6 h.^50^
All-cause re-hospitalisation	Unscheduled admission to hospital for any reason, defined as a being admitted for more than 24 h or past a calendar day.^6,20^
Bleeding events	Type 1: bleeding that is not actionable and does not cause the patient to seek unscheduled performance of studies, hospitalization, or treatment by a healthcare professional. Type 2: any clinically overt sign of haemorrhage that is actionable but does not meet criteria for Type 3, Type 4 (coronary artery bypass graft surgery [CABG]-related), or Type 5 (fatal bleeding) bleeding. The bleeding must require diagnostic studies, hospitalization, or treatment by a healthcare professional. In particular, the bleeding must meet at least one of the following criteria: Requires intervention, defined as a healthcare professional–guided medical treatment or percutaneous intervention to stop or treat bleeding, including temporarily or permanently discontinuing a medication or study drug. Bleeding leads to hospitalization or an increased level of care, defined as leading to or prolonging hospitalization or transfer to a hospital unit capable of providing a higher level of care. The bleeding prompts evaluation, defined as leading to an unscheduled visit to a healthcare professional resulting in diagnostic testing (laboratory or imaging). Type 3a: any transfusion with overt bleeding; overt bleeding plus haemoglobin drop ≥3 to <5 g/dL (provided haemoglobin drop is related to bleeding). Type 3b: overt bleeding plus haemoglobin drop ≥5 g/dL (provided haemoglobin drop is related to bleed); cardiac tamponade; bleeding requiring surgical intervention for control (excluding dental/nasal/skin/haemorrhoid); bleeding requiring intravenous vasoactive drugs. Type 3c: intracranial haemorrhage; sub-categories confirmed by autopsy or imaging, or lumbar puncture; intraocular bleed compromising vision. Type 4: CABG–related bleeding; peri-operative intracranial bleeding within 48 h; re-operation after closure of sternotomy for the purpose of controlling bleeding; transfusion of ≥5 units of whole blood or packed red blood cells within a 48-h period; chest tube output ≥2 L within a 24-h period. Type 5: fatal bleeding.^22^
Cardiac arrest	Cardiac arrest is defined as a verified sudden cessation of cardiac mechanical activity causing unresponsiveness, absence of normal breathing and no signs of circulation (excluding syncope or profound vagally mediated bradycardia) with ventricular fibrillation, rapid ventricular tachycardia or bradycardia resulting in loss of consciousness, pulseless electrical activity, or asystole as the major causes. Return of spontaneous circulation (ROSC) is defined as the resumption of a sustained heart rhythm that perfuses the body after cardiac arrest. Signs include a palpable pulse, measurable blood pressure and/or respiratory effort.^51^
Cardiogenic shock	Cardiogenic shock is defined as any one of the following: (1) ‘beginning’ cardiogenic shock or compensated shock where a patient may be volume overloaded, tachycardic, and/or hypotensive but no evidence of hypoperfusion on physical exam or laboratory studies. It also includes patients with a (2) ‘classic’ cardiogenic shock with evidence of hypoperfusion on physical exam and laboratory studies ‘cold and wet.’ Invasive haemodynamics (if available) demonstrate the classic depressed cardiac index associated with cardiogenic shock. Cardiogenic shock also includes patients with (3) ‘deteriorating’ and includes above patients plus failure of initial interventions in restoring adequate perfusion in 30 min and further escalation is required. Cardiogenic shock also includes (4) ‘escalation’ cardiogenic shock which is an increase in the number or intensity of intravenous therapies to address hypoperfusion, or addition of mechanical circulatory support after the initial 30-minute period of observation and treatment. It can also include patients who are highly unstable, often with circulatory collapse and/or refractory cardiac arrest with ongoing CPR. They are being supported by multiple simultaneous acute interventions including ECMO-facilitated CPR (eCPR).^52^
Cause-specific hospitalisation	Unscheduled hospitalisation due to either cardiovascular or non-cardiovascular causes. Unscheduled hospitalisation is defined as a being admitted for more than 24 h or past a calendar day. Cardiovascular causes include conditions such as HF, cardiogenic shock, bioprosthetic or native valve dysfunction, myocardial infarction, stroke, thromboembolism, bleeding, tamponade, vascular complication, arrhythmia or conduction system disturbances, cardiovascular infection (e.g. mediastinitis, endocarditis), or other clear cardiovascular cause. Non-cardiovascular cause includes (but is not limited to) respiratory failure not related to cardiovascular disease (e.g. pneumonia), renal failure, liver failure, infection (e.g. urosepsis), cancer, trauma, and suicide.^6,53^
DVT	DVT is the formation of a thrombus (blood clot) in a deep vein, usually in the legs, but may also include the arms, which partially or completely obstructs blood flow.^54^
Device implantation	Implantation of any of: Transvenous permanent pacemaker is an electronic device that is implanted in the subcutaneous tissue and gives the heart an electrical stimulation through transvenous wires. Leadless pacemaker is an electronic device that is implanted directly into the right ventricle. Transvenous ICD is a device that is used to correct abnormal heartbeat through transvenous wires. Subcutaneous ICD is an ICD with a presternal lead and is positioned between the latissimus dorsi and serratus muscle within the subcutaneous tissue. Extravascular ICD is an ICD with a substernal lead and the device in the subcutaneous tissue of the lateral thorax. CRT device and pacemaker (CRT-P) is defined as a biventricular pacemaker that sends electrical stimulation to both ventricles. CRT-D is a biventricular pacemaker and defibrillator.^55,56^
Heart transplant	Surgery in which a failing, diseased heart is replaced with a donor heart.^57^
Hospitalized ventricular tachycardia	The patient was hospitalized with ventricular tachycardia, defined as ≥3 consecutive beats with a rate >100 beats per minute originating from the ventricles, independent from atrial and atrioventricular (AV) nodal conduction.^46^
ICD therapy delivery	Delivery of either an ICD shock or antitachycardia pacing.^55^
Mechanical circulatory support	Use of mechanical circulatory support devices, such as left ventricular assist device.
AF or AFL	AF is defined as a supraventricular tachyarrhythmia with uncoordinated atrial electrical activation and consequently ineffective atrial contraction. The minimum duration of an ECG tracing of AF required to establish the diagnosis of clinical AF is at least 30 s, or entire 12-lead ECG. AFL is defined as a supraventricular tachyarrhythmia with co-ordinated but overly rapid atrial electrical activation, usually with some degree of AV node conduction block. The minimum duration of an ECG tracing of AF required to establish the diagnosis of clinical AFL is at least 30 s, or entire 12-lead ECG.^58^
Pulmonary embolism	A condition in which one or more emboli, usually arising from a thrombus formed in the veins, are lodged in and obstruct the pulmonary arterial system.^59^
Systemic embolism	Systemic embolism is defined as a hospital encounter with a principal diagnosis of an arterial embolism and thrombosis,^60^ excluding stroke or transient ischaemic attack.
Transient ischaemic attack	Transient ischaemic attack is a transient focal neurological signs or symptoms lasting <24 h presumed to be due to focal brain, spinal cord, or retinal ischaemia, but without evidence of acute infarction by neuroimaging or pathology, or with no imaging performed.^61^
Unplanned cardiac surgery	Unplanned cardiac surgery is defined as an unplanned surgical intervention to the heart and the great vessels.^62^
Worsening HF	HF is a clinical syndrome characterized by typical symptoms (e.g. dyspnoea) and/or signs (e.g. ankle swelling), caused by a structural and/or functional cardiac abnormality (e.g. left ventricular hypertrophy or impairment), and associated with elevated natriuretic peptide levels and/or objective evidence of pulmonary or systemic congestion from a cardiogenic origin at rest or with exercise. Worsening HF is defined as either an unplanned HF hospitalisation or urgent outpatient visit for HF. Unplanned HF hospitalisation is defined as a patient requiring an unscheduled hospital admission for a *primary diagnosis* of HF with a length of stay that either exceeds 24 h or crosses a calendar day (if hospital admission and discharge times are unavailable). To satisfy the criteria for a worsening HF event, the patient must have an urgent, unscheduled office or emergency visit for HF with signs, symptoms, and diagnostic testing results identical to those already described above. The patient must also require treatment for HF such as significant dose increase of oral diuretics, intravenous diuretics or mechanical or surgical intervention for HF. Importantly, clinic visits for scheduled administration of HF therapies or procedures (e.g. intravenous diuretics, intravenous vasoactive agents, or mechanical fluid removal) do not qualify as non-hospitalized HF events.^6,49,63^

Abbreviations: AF, atrial fibrillation; AFL, atrial flutter; CABG, coronary artery bypass; CPR, cardiopulmonary resuscitation; CRT, cardiac resynchronisation therapy; DVT, deep vein thrombosis; ECG, electrocardiogram; HF, heart failure; ICD, implantable cardioverter defibrillator.

### Generic domain: Level 2 variables

For the generic cardiovascular outcome measures domain, 18 Level 2 variables were agreed: acute kidney injury, all-cause re-hospitalisation, bleeding events, cardiac arrest, cardiogenic shock, cause-specific hospitalisation, deep vein thrombosis (DVT), device implantation (including transvenous pacemaker, leadless pacemaker, transvenous implantable cardioverter defibrillator [ICD], subcutaneous ICD, cardiac-resynchronisation therapy pacemaker or defibrillator [CRT-P, CRT-D]), heart transplant, hospitalized ventricular tachycardia, ICD therapy delivery (e.g. cardioversion or antitachycardia therapy), mechanical circulatory support, atrial fibrillation/flutter (AF), pulmonary embolism, systemic embolism, transient ischaemic attack, unplanned cardiac surgery, and worsening HF (*Table 1*).

### Acute coronary syndrome/percutaneous coronary intervention domain: Level 1 variables

For the ACS/PCI outcome measures domain, eight Level 1 variables were agreed: acute kidney injury requiring renal replacement therapy, cardiac arrest, HF hospitalisation, major bleeding event, target lesion stent thrombosis, target vessel related myocardial infarction, unplanned target-vessel coronary artery bypass graft (CABG) surgery, and unplanned target-vessel PCI (*Table 2*).

**Table 2:** Acute coronary syndrome/percutaneous coronary intervention—clinical outcomes and their definitions

**Acute coronary syndrome/PCI: Level 1 variables**	
Acute kidney injury requiring renal replacement therapy	Renal replacement therapy includes ultrafiltration (haemofiltration), haemodialysis or peritoneal dialysis.^50^
Cardiac arrest	Cardiac arrest is defined as a verified sudden cessation of cardiac activity causing unresponsiveness, absence of normal breathing and no signs of circulation (excluding syncope or profound vagally mediated bradycardia) with ventricular fibrillation, rapid ventricular tachycardia or bradycardia resulting in loss of consciousness, pulseless electrical activity, or asystole as the major causes. ROSC is defined as the resumption of a sustained heart rhythm that perfuses the body after cardiac arrest. Signs include a palpable pulse, measurable blood pressure and/or respiratory effort.^51^
Heart failure hospitalisation	Hospital admission primarily due to heart failure. Heart failure is a clinical syndrome characterized by typical symptoms (e.g. dyspnoea) and/or signs (e.g. ankle swelling), caused by a structural and/or functional cardiac abnormality (e.g. left ventricular hypertrophy or impairment), and associated with elevated natriuretic peptide levels and/or objective evidence of pulmonary or systemic congestion from a cardiogenic origin at rest or with exercise. Unplanned HF hospitalisation is defined as a patient requiring an unscheduled hospital admission for a *primary diagnosis* of HF with a length of stay that either exceeds 24 h or crosses a calendar day (if hospital admission and discharge times are unavailable). To satisfy the criteria for a HF hospitalisation, the patient must be admitted primarily for HF with signs, symptoms, and diagnostic testing results identical to those already described above. The patient must also require treatment for HF such as significant augmentation of oral diuretics, intravenous diuretics, or mechanical or surgical intervention for HF.^6,49,53,63^ Please record worsening heart failure under the Level 2 section of the generic outcomes, if available.
Major bleeding event	Type 2: any clinically overt sign of haemorrhage that is actionable but does not meet criteria for Type 3, Type 4 (coronary artery bypass graft surgery [CABG]-related), or Type 5 (fatal bleeding) bleeding. The bleeding must require diagnostic studies, hospitalization, or treatment by a healthcare professional. In particular, the bleeding must meet at least one of the following criteria: Requires intervention, defined as a healthcare professional–guided medical treatment or percutaneous intervention to stop or treat bleeding, including temporarily or permanently discontinuing a medication or study drug. Bleeding leads to hospitalization or an increased level of care, defined as leading to or prolonging hospitalization or transfer to a hospital unit capable of providing a higher level of care. The bleeding prompts evaluation, defined as leading to an unscheduled visit to a healthcare professional resulting in diagnostic testing (laboratory or imaging). Type 3a: any transfusion with overt bleeding; overt bleeding plus haemoglobin drop ≥3 to <5 g/dL (provided haemoglobin drop is related to bleeding). Type 3b: overt bleeding plus haemoglobin drop ≥5 g/dL (provided haemoglobin drop is related to bleed); cardiac tamponade; bleeding requiring surgical intervention for control (excluding dental/nasal/skin/haemorrhoid); bleeding requiring intravenous vasoactive drugs. Type 3c: intracranial haemorrhage; sub-categories confirmed by autopsy or imaging, or lumbar puncture; intraocular bleed compromising vision. Type 4: CABG–related bleeding; peri-operative intracranial bleeding within 48 h; reoperation after closure of sternotomy for the purpose of controlling bleeding; transfusion of ≥5 units of whole blood or packed red blood cells within a 48-h period; chest tube output ≥2 L within a 24-h period. Type 5: fatal bleeding.^22^ Note: Major bleeding includes fatal bleeding events, symptomatic bleeding in a critical areas or organs (e.g. intracranial, intraspinal, intraocular, retroperitoneal, intra-articular or peri-cardial, or intramuscular with compartment syndrome), and/or fall in haemoglobin level of ≥20 g/L (≥2 g/dL) or transfusion of ≥2 units of blood. In-hospital major bleeding does not include CABG-related bleeding. Fatal bleeding should be selected when bleeding is believed to be the primary cause of death. Intracranial haemorrhage, of any severity, should ideally be confirmed by scanning. Other major bleeding should only be selected if the patient had a major bleeding episode other than those stated above.^21^
Target lesion stent thrombosis	Target lesion stent thrombosis is defined as occurring when clinical presentation is consistent with acute coronary syndrome of a previously treated lesion. The categories are defined as follows: Definite is defined as angiographic confirmation of stent/scaffold thrombosis, the presence of a thrombus that originates in the stent/scaffold or in the segment 5 mm proximal or distal to the stent/scaffold or in a side branch originating from the stented/scaffolded segment and the presence of at least one of the following criteria: Acute onset of ischaemic symptoms at rest New electrocardiographic changes suggestive of acute ischaemia Typical rise and fall in cardiac biomarkers (refer to definition of spontaneous myocardial infarction) Pathological confirmation of stent/scaffold thrombosis Evidence of recent thrombus within the stent/scaffold determined at autopsy. Examination of tissue retrieved following thrombectomy (visual/histology) Probable is defined, regardless of the time after the index procedure, as any myocardial infarction that is related to documented acute ischaemia in the territory of the implanted stent/scaffold without angiographic confirmation of stent/scaffold thrombosis and in the absence of any other obvious cause. Silent is defined as the incidental angiographic documentation of stent occlusion in the absence of clinical signs or symptoms is not considered stent thrombosis.^64^
Target vessel related myocardial infarction	Target vessel myocardial infarction is defined as myocardial necrosis in the vascular territory of a previously treated target vessel. As well as direct evidence of invasive angiography, electrocardiographic or other imaging evidence such as echocardiography (e.g. newly developed regional wall motion abnormality or extension of previous abnormality) can be used to adjudicate the involvement of the target vessel territory. The target vessel was defined as the entire major coronary vessel or bypass graft proximal and distal to the target lesion including upstream and downstream branches and the target lesion itself. The left main coronary artery and any vessel originating from the left main coronary artery, or its major branches is, defined as target vessel.^47,64^
Unplanned target-vessel coronary artery bypass graft surgery	Repeat revascularisation of the target vessel with unplanned coronary artery bypass grafting (CABG) surgery after the index procedure. Target vessel CABG is defined as any CABG of any segment of the target vessel including the target lesion. The target vessel was defined as the entire major coronary vessel or bypass graft proximal and distal to the target lesion including upstream and downstream branches and the target lesion itself. The left main coronary artery and any vessel originating from the left main coronary artery, or its major branches are, defined as target vessel. CABG is a procedure to bypass diseased segment(s) of the coronary tree using blood vessels derived other parts of the body and connected to the aorta.^64,65^
Unplanned target-vessel PCI	An unplanned PCI after the index procedure. Repeat target vessel PCI is defined as any repeat PCI of any segment of the target vessel including the target lesion. The target vessel is defined as the entire major coronary vessel or bypass graft proximal and distal to the target lesion including upstream and downstream branches and the target lesion itself. The left main coronary artery and any vessel originating from the left main coronary artery, or its major branches are, defined as target vessel. PCI is defined as the placement of an angioplasty guidewire, balloon, or other device (e.g. stent, atherectomy, brachytherapy, or thrombectomy catheter) into a native coronary artery or a graft for the purpose of mechanical coronary revascularisation. The assessment of coronary lesion severity by fluoroscopy, intracoronary imaging (e.g. intravascular ultrasonography) or physiology (e.g. fractional flow reserve) is not considered a PCI procedure.^64,65^
**Acute coronary syndrome/PCI: Level 2 variables**	
Attendance at cardiac rehabilitation	Cardiovascular rehabilitation is a multi-factorial and comprehensive intervention in secondary prevention, supervised and carried out by adequately trained health professionals.^66^
Cardiovascular hospitalisation	Unscheduled hospitalized primarily due to cardiovascular disease. Unscheduled hospitalisation is defined as a being admitted for more than 24 h or past a calendar day due to primarily a cardiovascular condition. Cardiovascular causes include conditions such as heart failure, cardiogenic shock, bioprosthetic or native valve dysfunction, myocardial infarction, stroke, thromboembolism, bleeding, tamponade, vascular complication, arrhythmia or conduction system disturbances, cardiovascular infection (e.g. mediastinitis, endocarditis), or other clear cardiovascular cause.^6,53^
CCS angina class	CCS Grade I: Ordinary physical activity does not cause angina, such as walking and climbing stairs. Angina with strenuous or rapid or prolonged exertion at work or recreation. CCS Grade II: Slight limitation of ordinary activity. Walking or climbing stairs rapidly, walking uphill, walking or stair climbing after meals, or in cold, or in wind, or under emotional stress, or only during the few hours after awakening. Walking more than two blocks on the level and climbing more than one flight of ordinary stairs at a normal pace and in normal conditions. CCS Grade III: Marked limitation of ordinary physical activity. Walking one or two blocks on the level and climbing one flight of stairs in normal conditions and at normal pace. CCS Grade IV: Inability to carry on any physical activity without discomfort, anginal syndrome may be present at rest.^67^
Hospitalisation with unstable angina	Unscheduled admission to hospital with unstable angina as the primary cause. Unstable angina is defined as myocardial ischaemia at rest or on minimal exertion in the absence of acute cardiomyocyte injury/necrosis using high-sensitive (hs)-cardiac troponin (cTn). Unscheduled hospitalisation is defined as a being admitted for more than 24 h or past a calendar day due to primarily a cardiovascular condition.^6,53,68^
Left ventricular ejection fraction	Ejection fraction is ideally measured with echocardiography for consistency.
Minor bleeding event	Bleeding that is not actionable and does not cause the patient to seek unscheduled performance of studies, hospitalisation, or treatment by a healthcare professional; may include episodes leading to self-discontinuation of medical therapy by the patient without consulting a healthcare professional (Bleeding Academic Research Consortium Type 1).^21^
Vascular access complication	Access site haematoma, arteriovenous fistula, peripheral ischaemia, peripheral nerve injury, pseudoaneurysm, or retroperitoneal haemorrhage. Major access-related non-vascular events are defined as one of the following: Non-vascular structure, non-cardiac structured perforation, injury, or infection resulting in death, BARC type ≥3 bleeding, irreversible nerve injury or requiring unplanned surgery or percutaneous intervention. Non-vascular access site (e.g. trans-apical left ventricular) perforation, injury, or infection resulting in death, BARC type ≥3 bleeding, irreversible nerve injury or requiring unplanned surgery or percutaneous intervention. Minor access-related non-vascular events are defined as one of the following: Non-vascular structure, non-cardiac structured perforation, injury, or infection not resulting in death, BARC type ≥3, irreversible nerve injury, or requiring unplanned surgery or percutaneous intervention. Non-vascular access site (e.g. trans-apical left ventricular) perforation, injury, or infection not resulting in death, BARC type ≥3 bleeding, irreversible nerve injury or requiring unplanned surgery or percutaneous intervention.^21,53^

Abbreviations: BARC, Bleeding Academic Research Consortium; CABG, coronary artery bypass; CCS, Canadian Cardiovascular Society; PCI, percutaneous coronary intervention.

### Acute coronary syndrome/percutaneous coronary intervention domain: Level 2 variables

For the ACS/PCI outcome measures domain, seven Level 2 variables were agreed: attendance at cardiac rehabilitation, cardiovascular hospitalisation, Canadian Cardiovascular Society (CCS) angina class, hospitalisation with unstable angina, left ventricular ejection fraction, minor bleeding event, and vascular access complication (*Table 2*).

### Atrial fibrillation domain: Level 1 variables

For the AF outcome measures domain, two Level 1 variables were agreed: cardiovascular hospitalisation, and catheter ablation (*Table 3*).

**Table 3:** Atrial fibrillation—clinical outcomes and their definitions

**AF: Level 1 variables**	
Cardiovascular hospitalisation	Admission to hospital primarily due to cardiovascular disease. Unscheduled hospitalisation is defined as a being admitted for more than 24 h or past a calendar day due to primarily a cardiovascular condition. CV causes include conditions such as heart failure, cardiogenic shock, bioprosthetic or native valve dysfunction, myocardial infarction, stroke, thromboembolism, bleeding, tamponade, vascular complication, arrhythmia or conduction system disturbances, cardiovascular infection (e.g. mediastinitis, endocarditis), or other clear cardiovascular cause.^6,53^
Catheter ablation	Catheter ablation for AF or AFL is defined as a procedure in which catheters are inserted through the veins or arteries to the heart, and energy (e.g. radiofrequency, cryoablation) is delivered to prevent propagation of abnormal AF or AFL. AF is defined as a supraventricular tachyarrhythmia with uncoordinated atrial electrical activation and consequently ineffective atrial contraction. The minimum duration of an ECG tracing of AF required to establish the diagnosis of clinical AF is at least 30 s, or entire 12-lead ECG. AFL is defined as a supraventricular tachyarrhythmia with co-ordinated but overly rapid atrial electrical activation, usually with some degree of AV node conduction block. The minimum duration of an ECG tracing of AFL required to establish the diagnosis of clinical AFL is at least 30 s, or entire 12-lead ECG.^17^
**Atrial fibrillation: Level 2 variables**	
All-cause hospitalisation	Unscheduled admission to hospital for any reason. Hospitalisation is defined as a being admitted for more than 24 h or past a calendar day.^6,53^
Burden of atrial fibrillation	Burden is defined as the amount of time spent in atrial fibrillation as a proportion of the total monitoring period. Monitoring can be in the form of invasive and non-invasive monitoring devices. Duration of the device monitoring period is the fixed monitoring period using ambulatory and between downloads of invasive monitoring devices.^58^
Cardioversion	Electrical cardioversion (external or internal) is defined as a procedure in which direct current is used to restore sinus rhythm. Pharmacologic cardioversion is defined as a procedure in which antiarrhythmic medications are used to restore sinus rhythm.^58^
Device implantation	Implantation of: Transvenous permanent pacemaker is an electronic device that is implanted in the subcutaneous tissue and gives the heart an electrical stimulation through transvenous wires. Leadless pacemaker is an electronic device that is implanted directly into the right ventricle. ICD, a device that is used to correct abnormal heartbeat with transvenous wires. Subcutaneous ICD is an ICD with a presternal lead and is positioned between the latissimus dorsi and serratus muscle within the subcutaneous tissue. Extravascular ICD is an ICD with a substernal lead and the device in the subcutaneous tissue of the lateral thorax. CRT device and pacemaker (CRT-P) is defined as a biventricular pacemaker that sends electrical stimulation to both ventricles. CRT-D is a biventricular pacemaker and defibrillator.^55,56^
Implantable monitoring device	An implantable device that allows remote rhythm monitoring.^55^
Recurrence of AF	Recurrence of atrial fibrillation/flutter. AF is defined as a supraventricular tachyarrhythmia with uncoordinated atrial electrical activation and consequently ineffective atrial contraction. The minimum duration of an ECG tracing of AF required to establish the diagnosis of clinical AF is at least 30 s, or entire 12-lead ECG. AFL is defined as a supraventricular tachyarrhythmia with co-ordinated but overly rapid atrial electrical activation, usually with some degree of AV node conduction block. The minimum duration of an ECG tracing of AFL required to establish the diagnosis of clinical AFL is at least 30 s, or entire 12-lead ECG.^17^

Abbreviations: AF, atrial fibrillation; AFL, atrial flutter; CRT, cardiac resynchronisation therapy; ECG, electrocardiogram; ICD, implantable cardioverter defibrillator.

### Atrial fibrillation domain: Level 2 variables

For the AF outcome measures domain, six Level 2 variables were agreed: all-cause hospitalisation, burden of AF (time spent in AF out of total monitoring period), cardioversion, device implantation (e.g. pacemaker, CRT, and ICD), implantable monitoring device, and recurrence of AF (*Table 3*).

### Heart failure domain: Level 1 variables

For the HF outcome measures domain, five Level 1 variables were agreed: all-cause hospitalisation, HF re-hospitalisation, heart transplantation, left ventricular ejection fraction, and implant of a left ventricular assist device (*Table 4*).

**Table 4:** Heart failure—clinical outcomes and their definitions

**Heart failure: Level 1 variables**	
All-cause re-hospitalisation	Unscheduled hospitalisation for any cause, defined as a being admitted for more than 24 h or past a calendar day.^6,53^
Heart failure re-hospitalisation	Hospital admission primarily related to heart failure. Heart failure is a clinical syndrome characterized by typical symptoms (e.g. dyspnoea) and/or signs (e.g. ankle swelling), caused by a structural and/or functional cardiac abnormality (e.g. left ventricular hypertrophy or impairment), and associated with elevated natriuretic peptide levels and/or objective evidence of pulmonary or systemic congestion from a cardiogenic origin at rest or with exercise. Unplanned HF hospitalisation is defined as a patient requiring an unscheduled hospital admission for a *primary diagnosis* of HF with a length of stay that either exceeds 24 h or crosses a calendar day (if hospital admission and discharge times are unavailable). To satisfy the criteria for a HF hospitalisation, the patient must be admitted primarily for HF with signs, symptoms, and diagnostic testing results identical to those already described above. The patient must also require treatment for HF such as significant augmentation of oral diuretics, intravenous diuretics, or mechanical or surgical intervention for HF.^6,49,53,63^
Heart transplantation	Receipt of surgery in which a failing, diseased heart is replaced with a healthier donor heart.^57^
Left ventricular assist device	Implant of a left ventricular assist device.
Left ventricular ejection fraction	Ejection fraction, ideally measured with echocardiography.
**Heart failure domain: Level 2 variables**	
Device implantation	Implantation of: Transvenous permanent pacemaker is an electronic device that is implanted in the subcutaneous tissue and gives the heart an electrical stimulation through transvenous wires. Leadless pacemaker is an electronic device that is implanted directly into the right ventricle. Transvenous ICD is a device that is used to correct abnormal heartbeat through transvenous wires. Subcutaneous ICD is an ICD with a presternal lead and is positioned between the latissimus dorsi and serratus muscle within the subcutaneous tissue. Extravascular ICD is an ICD with a substernal lead and the device in the subcutaneous tissue of the lateral thorax. CRT device and pacemaker (CRT-P) is defined as a biventricular pacemaker that sends electrical stimulation to both ventricles. CRT-D is a biventricular pacemaker and defibrillator.^55,56^
Resuscitated ventricular tachyarrhythmia	The patient was successfully resuscitated and had ROSC from a ventricular tachyarrhythmia.

Abbreviations: CRT, cardiac resynchronisation therapy; HF, heart failure; ICD, implantable cardioverter defibrillator; NYHA, New York Heart Association; ROSC, return of spontaneous circulation.

### Heart failure domain: Level 2 variables

For the HF outcome measures domain, two Level 2 variables were agreed: device implant (e.g. pacemaker, CRT, and ICD), and resuscitated ventricular arrhythmia (*Table 4*). Notably, the HF Working Group advised that additional parameters would be advantageous for monitoring the chronic disease management aspects of HF; these are discussed in detail in a separate publication.^23^

### Transcatheter aortic valve intervention domain: Level 1 variables

For the TAVI outcome measures domain, four Level 1 variables were agreed: aortic regurgitation, device implantation (e.g. pacemaker, CRT, and ICD), New York Heart Association class, and re-intervention on the aortic valve (*Table 5*).

**Table 5:** Transcatheter aortic valve intervention—clinical outcomes and their definitions

**Transcatheter aortic valve intervention—level 1 variables**	
Aortic regurgitation	Presence of aortic regurgitation, and severity (mild/moderate/severe) as determined by echocardiography based on Doppler parameters according to the criteria of the VARC 3 criteria.^53^
Device implantation	Implantation of: Transvenous permanent pacemaker is an electronic device that is implanted in the subcutaneous tissue and gives the heart an electrical stimulation through transvenous wires. Leadless pacemaker is an electronic device that is implanted directly into the right ventricle. Transvenous ICD is a device that is used to correct abnormal heartbeat through transvenous wires. Subcutaneous ICD is an ICD with a presternal lead and is positioned between the latissimus dorsi and serratus muscle within the subcutaneous tissue. Extravascular ICD is an ICD with a substernal lead and the device in the subcutaneous tissue of the lateral thorax. CRT device and pacemaker (CRT-P) is defined as a biventricular pacemaker that sends electrical stimulation to both ventricles. CRT-D is a biventricular pacemaker and defibrillator.^55,56^
NYHA class	NYHA class I: no limitations of physical activity. Ordinary physical activity does not cause undue fatigue, palpitations, or dyspnoea. NYHA class II: slight limitation of physical activity. The patient is comfortable at rest. Ordinary physical activity results in fatigue, palpitations, or dyspnoea. NYHA class III: marked limitation of physical activity. The patient is comfortable at rest. Less than ordinary activity causes fatigue, palpitations, or dyspnoea. NYHA class IV: inability to carry on any physical activity without discomfort. Heart failure symptoms are present even at rest or with minimal exertion.^49,63^
Re-intervention on the aortic valve:	Re-do TAVI is a different procedure to the index TAVI, and a separate registration form should be completed. Balloon aortic valvuloplasty is a transcatheter balloon dilatation of the implanted aortic valve after the completion of the index procedure. Surgical aortic valve replacement is defined as a deployment of a new (mechanical or bioprosthetic) aortic valve surgically. Other aortic valve surgery is any other surgical intervention on the aortic valve.
**Transcatheter aortic valve intervention—level 2 variables**	
Access-related non-vascular complications	Major access-related non-vascular events are defined as one of the following: Non-vascular structure, non-cardiac structured perforation, injury, or infection resulting in death, BARC type ≥3 bleeding, irreversible nerve injury or requiring unplanned surgery or percutaneous intervention. Non-vascular access site (e.g. trans-apical left ventricular) perforation, injury, or infection resulting in death, BARC type ≥3 bleeding, irreversible nerve injury or requiring unplanned surgery or percutaneous intervention. Minor access-related non-vascular events are defined as one of the following: Non-vascular structure, non-cardiac structured perforation, injury, or infection not resulting in death, BARC type ≥ 3 bleeding, irreversible nerve injury, or requiring unplanned surgery or percutaneous intervention. Non-vascular access site (e.g. trans-apical left ventricular) perforation, injury, or infection not resulting in death, BARC type ≥3 bleeding, irreversible nerve injury or requiring unplanned surgery or percutaneous intervention.^53^
All-cause re-hospitalisation	Unscheduled hospital admission for any reason. Unscheduled hospitalisation is defined as a being admitted for more than 24 h or past a calendar day.^6,53^
Cardiac arrest	Cardiac arrest is defined as a verified sudden cessation of cardiac activity causing unresponsiveness, absence of normal breathing and no signs of circulation (excluding syncope or profound vagally mediated bradycardia) with ventricular fibrillation, rapid ventricular tachycardia, or bradycardia resulting in loss of consciousness, pulseless electrical activity, or asystole as the major causes. ROCS is defined as the resumption of a sustained heart rhythm that perfuses the body after cardiac arrest. Signs include a palpable pulse, measurable blood pressure, and/or respiratory effort.^51^
Cardiogenic shock	Cardiogenic shock is defined as any one of the following: (1) ‘beginning’ cardiogenic shock or compensated shock where a patient may be volume overloaded, tachycardic, and/or hypotensive but no evidence of hypoperfusion on physical exam or laboratory studies. It also includes patients with a (2) ‘classic’ cardiogenic shock with evidence of hypoperfusion on physical exam and laboratory studies ‘cold and wet.’ Invasive haemodynamics (if available) demonstrate the classic depressed cardiac index associated with cardiogenic shock. Cardiogenic shock also includes patients with (3) ‘deteriorating’ and includes above patients plus failure of initial interventions in restoring adequate perfusion in 30 min and further escalation is required. Cardiogenic shock also includes (4) ‘escalation’ cardiogenic shock in which an increase in the number or intensity of intravenous therapies to address hypoperfusion, or addition of mechanical circulatory support after the initial 30-min period of observation and treatment. It can also include patients who are highly unstable, often with circulatory collapse and/or refractory cardiac arrest with ongoing CPR. They are being supported by multiple simultaneous acute interventions including ECMO-facilitated CPR (eCPR).^52^
CCS angina status	CCS Grade I: ordinary physical activity does not cause angina, such as walking and climbing stairs. Angina with strenuous or rapid or prolonged exertion at work or recreation. CCS Grade II: slight limitation of ordinary activity. Walking or climbing stairs rapidly, walking uphill, walking or stair climbing after meals, or in cold, or in wind, or under emotional stress, or only during the few hours after awakening. Walking more than two blocks on the level and climbing more than one flight of ordinary stairs at a normal pace and in normal conditions. CCS Grade III: marked limitation of ordinary physical activity. Walking one or two blocks on the level and climbing one flight of stairs in normal conditions and at normal pace. CCS Grade IV: inability to carry on any physical activity without discomfort, anginal syndrome may be present at rest.^67^
CABG surgery	CABG surgery after the TAVI procedure. CABG is a procedure to bypass diseased segment(s) of the coronary tree using blood vessels derived from other parts of the body and connected to the aorta.^62^
Creatinine	Serum creatinine assay, in µmol/L.
Endocarditis	Infective endocarditis is diagnosed if at least one of the following criteria is met: (1) Fulfilment of the Duke criteria for endocarditis (2) Evidence of abscess, pus, or vegetation confirmed as secondary to infection by histological or microbiological studies during re-operation; and (3) Evidence of abscess, pus, or vegetation confirmed on autopsy.^53^
Left ventricular ejection fraction	Ejection fraction, ideally measured with echocardiography.
AF or AFL	A new diagnosis of AF or AFL. AF is defined as a supraventricular tachyarrhythmia with uncoordinated atrial electrical activation and consequently ineffective atrial contraction. The minimum duration of an ECG tracing of AF required to establish the diagnosis of clinical AF is at least 30 s, or entire 12-lead ECG. AFL is defined as a supraventricular tachyarrhythmia with co-ordinated but overly rapid atrial electrical activation, usually with some degree of atrioventricular node conduction block. The minimum duration of an ECG tracing of AFL required to establish the diagnosis of clinical AFL is at least 30 s, or entire 12-lead ECG.^58^
Renal replacement therapy	The patient developed a new requirement for renal replacement therapy. Renal replacement therapy includes ultrafiltration (haemofiltration), haemodialysis, or peritoneal dialysis.^50^
Other transcatheter heart valve procedure	Valve intervention after the index TAVI procedure, excluding repeat aortic valve intervention.
PCI	PCI after the TAVI procedure. PCI is the placement of an angioplasty guidewire, balloon, or other device (e.g. stent, atherectomy, brachytherapy, or thrombectomy catheter) into a native coronary artery or a graft for the purpose of mechanical coronary revascularisation. The assessment of the severity of a coronary lesion by fluoroscopy, intracoronary imaging (e.g. intravascular ultrasonography), or intracoronary physiology (e.g. fractional flow reserve) is not considered a PCI procedure.^65^
Residual aortic stenosis	Stage 1: Evidence of structural valve deterioration, non-structural valve dysfunction (other than paravalvular regurgitation or prosthesis-patient mismatch), thrombosis, or endocarditis without significant haemodynamic changes. Stage 2: Increase in mean trans valvular gradient >10 mm Hg resulting in mean gradient >20 mm Hg with concomitant decrease in effective orifice area (EOA) > 0.3 cm^2^ or > 25% and/or decrease in Doppler velocity index >0.1 or >20% compared with echocardiographic assessment performed 1–3 months post-procedure. Stage 3: Increase in mean trans valvular gradient >20 mm Hg resulting in mean gradient >30 mm Hg with concomitant decrease in EOA > 0.6 cm^2^ or > 50% and/or decrease in Doppler velocity index > 0.2 or > 40% compared with echocardiographic assessment performed 1–3 months post-procedure.^20^
Sternotomy/thoracotomy due to bleeding	The patient had a sternotomy/thoracotomy due to bleeding.

Abbreviations: AF, atrial fibrillation; AFL, atrial flutter; BARC, Bleeding Academic Research Consortium; CABG, coronary artery bypass; CCS, Canadian Cardiovascular Society; CPR, cardiopulmonary resuscitation; CRT, cardiac resynchronization therapy; ECG, electrocardiogram; ECMO, extracorporeal membrane oxygenation; ICD, implantable cardioverter defibrillator; NYHA, New York Heart Association; PCI, percutaneous coronary intervention; TAVI, transcatheter aortic valve intervention; VARC, Valve Academic Research Consortium.

### Transcatheter aortic valve intervention domain: Level 2 variables

For the TAVI outcome measures domain, 15 Level 2 variables were agreed: access-related non-vascular complications, all-cause re-hospitalisation, cardiac arrest, cardiogenic shock, CCS angina status, CABG surgery, creatinine, endocarditis, left ventricular ejection fraction, AF/flutter, renal replacement therapy, other transcatheter heart valve procedure, PCI, residual aortic stenosis, and sternotomy or thoracotomy due to bleeding (*Table 5*).

### Bleeding

Various classification systems for bleeding are used across the EuroHeart data standards,^14–17^ reflecting the relative strengths of each in specific in-patient settings. Of 59 respondents, the preferred bleeding classification for the outcome domain was BARC as selected by 25 (42%), with 18 (31%) voting for VARC, and 16 (27%) voting for ISTH as their first choice.

### Medical devices

Whenever a medical device featured within the outcomes domain is implanted or used, the associated unique device identification code will be recorded as a Level 1 variable to enable longitudinal device surveillance.

## Discussion

Following the ESC methodology for data standards development, we have derived and defined a suite of internationally agreed cardiovascular outcome measures. This catalogue spans ACS/PCI, AF, HF, and TAVI, and includes generic cardiovascular outcome measures that are applicable across a range of cardiovascular diseases. The cardiovascular outcome measures have been evaluated by international experts, and classified hierarchically as Level 1, meaning that collection of the variable is mandatory; or Level 2, where inclusion is optional and is based upon specific study goals. In total, we present 24 Level 1 and 48 Level 2 cardiovascular outcome measures.

The concept of standardized cardiovascular endpoints is not new. Similar work has been published, but is limited to specific cardiovascular diseases,^3–5^ or RCTs.^6^ Unlike previous frameworks, our cardiovascular outcome measure catalogue is purposefully designed to span many research study designs, as well as for the design of and utilisation in structured electronic health records. Differentiating between Level 1 and 2 variables means that centres may participate in data collection of only the ‘essentials’, and select additional optional variables to suit their specific needs. This will maximize the potential for implementation across heterogeneous healthcare environments with differing availability of electronic health records and central infrastructure to support data collection.

The EuroHeart initiative offers a toolset that is likely to enhance the quality and impact of cardiovascular healthcare research and patient care globally.^24^ Seemingly small differences in definitions may alter study conclusions, may potentially be misleading, and make comparisons between studies challenging.^25–27^ Indeed, regulatory authorities and most major clinical journals prefer prospective identification of a primary outcome measure with a robust statistical approach to multiplicity in outcomes.^28,29^ The alignment of these measures is necessary: the number of RCTs published is increasing year on year, and now exceeds 35 000 annually.^30^ In addition, researchers are employing novel methods by which to conduct RCTs and observational studies,^31^ including the use of routinely collected healthcare systems data and registry-based RCTs.^32^ Such use of structured data, not purposely designed for the research at hand, compels the use of clinical outcome measures selected from a finite range of variables,^6,33^ although we recognize that difficulties in determining whether the definition was met in individual cases, particularly where clinical data are incomplete.

Historically, outcomes such as myocardial infarction have limited specificity and sensitivity in administrative data.^34^ The ADAPTABLE trial [NCT02697916] used a common data model as the primary source of endpoint ascertainment without adjudication, and found that the positive predictive values for hospitalisation for myocardial infarction, stroke, and major bleeding, compared with adjudication were 90%, 72%, and 93%, respectively.^35^ Adjudication of outcome measures has traditionally been considered important to minimize noise and mitigate bias,^36^ however, recent evidence indicates that it may be supplemented or replaced by the use of routinely collected healthcare systems data for clinical outcome measure assessments.^36–39^ Similarly, HF re-hospitalisation rates within 30 days vary substantially depending on how they are measured—between 6.5% and 15% at 30 days according to a recent registry study.^40^ There is therefore a need to standardize the cardinal clinical outcome measures and their definitions to maximize the opportunities that are afforded by these developments in methodology to be fully realized.^13^ Additionally, in light of the increased use of composite outcomes in clinical trials,^41^ a consistent and universally agreed selection of the variables to be included in the design phase is important to minimize the risk of bias in these circumstances.^42^

### Strengths and limitations

EuroHeart provides an opportunity for co-ordinated collection and analysis of cardiovascular data across Europe and beyond. Within the first year of data collection, data were collected using internationally endorsed data standards for over 40 000 patients with ACS across seven countries.^14–17,43^ The present work extends and complements this, using an established and robust methodology,^18^ and harnessing the expertise of a wide range of international experts from diverse healthcare settings. However, we recognize the limitations of this work. In particular, the reliance on a select group of leading experts to define and classify outcome measures can introduce selection bias. The perspectives and experiences of these experts might not fully represent the diverse patient demographics or the full spectrum of clinical realities in different settings. This might limit the universality of the adopted measures. Although the importance of patient-reported outcomes measures (PROMs) and experiences (PREMs) is increasingly recognized,^44,45^ our remit for this project was limited to clinical outcomes.

This suite of outcomes may be used in clinical studies and for data alignment, and will be integrated into the EuroHeart IT platform to enable standardized measurement and federated research.

## Conclusion

We present a suite of internationally developed and prioritized outcome measures and their associated definitions for four common cardiovascular conditions, derived through an expert-led consensus process. These will be implemented within EuroHeart. Their consistent use is encouraged in other registries, healthcare systems data, RCTs, and observational research.

## Supplementary Material

ehae724_Supplementary_Data

## Notes

**Membership of the Global Cardiovascular Outcomes Consortium**: Victor Aboyans, Ana G. Almeida, Dan Atar, Gorav Batra, Antoni Bayés-Genís, Asad Bhatty, Giuseppe Biondi-Zoccai, Marc P. Bonaca, Nikolaos Bonaros, Bianca J.J.M. Brundel, Raffaele Bugiardini, Gianluca Campo, Barbara Casadei, Ruben Casado-Arroyo, Claudio Ceconi, Edina Cenko, Ovidiu Chioncel, Michele Ciccarelli, Louise Coats, Jean-Philippe Collet, Gheorghe-Andrei Dan, Victoria Delgado, Polychronis Dilaveris, Dobromir Dobrev, Erwan Donal, David Duncker, Sarah Moharem Elgamal, Justin A. Ezekowitz, Gregg Fonarow, Alan G. Fraser, Chris P. Gale, George Giannakoulas, Bruna Gigante, Massimiliano Gnecchi, Can Gollmann-Tepeköylü, Stephen J. Greene, Jordi Heijman, Jonathan Howes, Bernard Iung, Stefan James, Magnus T. Jensen, Vijay Kunadian, Malgorzata Lelonek, Sergio Leonardi, Erik Lerkevang Grove, Luca Liberale, Riccardo Liga, A. Michael Lincoff, Roberto Lorusso, Aldo P. Maggioni, Mamas Mamas, Olivia Manfrini, Fabio Mangiacapra, Nina Ajmone Marsan, María Martín-Fernandez, Jose L. Merino, Marco Metra, Alessandro Parolari, Cinzia Perrino, Lorenz Räber, Benyamin Rahmani, Peter P. Rainer, Giuseppe M.C. Rosano, Alexia Rossi, Andrea Rubboli, Tanja Rudolph, Sigrid Sandner, Gianluigi Savarese, Jolanta Siller-Matula, Samuel Sossalla, Cristiano Spadaccio, Eugenio Stabile, David Tanne, Jurrien ten Berg, Matthias Thielmann, Roderick W. Treskes, Izabella Uchmanowicz, Jacob A. Udell, Roland R.J. van Kimmenade, Lars Wallentin, Chris Wilkinson, and Marija Zdravkovic.

## Declarations

### Data Availability

The data underlying this article are available in the article and in its online supplementary material.

### Pre-registered Clinical Trial Number

None: this was not a clinical trial.
