# Supplementary material for: Definitions of clinical study outcome measures for cardiovascular diseases: the European Unified Registries for Heart Care Evaluation and Randomized Trials (EuroHeart)
Source: Eur Heart J. 2024 Nov 15;46(2):190–214. doi: 10.1093/eurheartj/ehae724 (PMC11704390; doi:10.1093/eurheartj/ehae724)
Supplement: ehae724_Supplementary_Data [file ehae724_supplementary_data.docx]

Derivation and definitions of clinical study outcome measures for cardiovascular diseases: the European Unified Registries for Heart Care Evaluation and Randomised Trials (EuroHeart)

**Supplementary data**

Supplementary table 1: Medline search strategy

| 1 | exp Heart Failure/ |
| --- | --- |
| 2 | ((heart or cardiac* or cardio* or myocardial* or diastolic* or systolic* or paroxysmal*) adj5 (failure* or edema* or oedema* or decompensation* of dyspnea* or asthma* or chronic* or insufficient*)).ti,ab,kf. |
| 3 | ((preserved ejection* or reduced ejection*) adj5 fraction*).ti,ab,kf. |
| 4 | (congestive heart* adj5 disease*).ti,ab,kf. |
| 5 | ((cardio renal* or cardiorenal* or reno cardiac* or renocardiac*) adj5 syndrome*).ti,ab,kf. |
| 6 | exp Arrhythmias, Cardiac/ |
| 7 | (arrhythmia* or dysrhythmia* or bradycardia* or bradyarrhythmia* or tachycardia* or tachyarrhythmia*).ti,ab,kf. |
| 8 | ((irregular* or slow* or rapid* or fast or junctional*) adj3 (heartbeat* or heart beat* or rhythm*)).ti,ab,kf. |
| 9 | ((atrial or auricular or ventricular) adj5 (fibrillation* or flutter*)).ti,ab,kf. |
| 10 | ((heart rhythm* or cardiac rhythm*) adj5 disorder*).ti,ab,kf. |
| 11 | (premature adj3 (atrial or ventricular or junctional or cardiac) adj3 (contraction* or complex*)).ti,ab,kf. |
| 12 | ((accelerat* or junctional*) adj5 rhythm*).ti,ab,kf. |
| 13 | (extra beats or heart block or heart blocks or AV block or AV blocks).ti,ab,kf. |
| 14 | Coronary Artery Disease/ |
| 15 | (atherosclerosis or atheroscleroses or arteriosclerosis or arterioscleroses or (coronary adj5 disease*)).ti,ab,kf. |
| 16 | (hard* adj3 arter*).ti,ab,kf. |
| 17 | (plaque adj4 build*).ti,ab,kf. |
| 18 | acute coronary syndrome/ |
| 19 | exp Myocardial Infarction/ |
| 20 | exp Percutaneous Coronary Intervention/ |
| 21 | Cardiac Rehabilitation/ |
| 22 | ((cardiac* or cardio* or heart*) adj5 (rehab* or conditioning*)).ti,ab,kf. |
| 23 | exp Aortic Valve/ |
| 24 | exp Aortic Valve Stenosis/ |
| 25 | exp Mitral Valve/ |
| 26 | exp mitral valve stenosis/ |
| 27 | exp mitral valve insufficiency/ |
| 28 | exp mitral valve regurgitation/ |
| 29 | Heart Valve Diseases/ |
| 30 | (left adj2 valv*).af. |
| 31 | (native adj2 valve*).af. |
| 32 | (mitral adj2 valv*).af. |
| 33 | (aortic adj2 valv*).af. |
| 34 | (valve adj2 (disease* or stenos* or insufficien*)).tw. |
| 35 | Heart Valve Prosthesis Implantation/ |
| 36 | Heart Valve Prosthesis/ |
| 37 | (valve adj2 (surg* or replace* or repair* or prosthe* or implant* or procedure*)).tw. |
| 38 | MitraClip.tw. |
| 39 | Transcatheter Aortic Valve Replacement/ |
| 40 | TAVI.tw. |
| 41 | pacemaker, artificial/ or cardiac resynchronization therapy devices/ |
| 42 | Defibrillators, Implantable/ |
| 43 | or/1-42 [cardiovascular diseases] |
| 44 | exp cohort studies/ |
| 45 | cohort$.tw. |
| 46 | controlled clinical trial.pt. |
| 47 | epidemiologic methods/ |
| 48 | limit 47 to yr=1966-1989 |
| 49 | exp case-control studies/ |
| 50 | (case$ and control$).tw. |
| 51 | or/44-46,48-50 |
| 52 | Randomized controlled trial.pt. |
| 53 | controlled clinical trial.pt. |
| 54 | randomized.ab. |
| 55 | placebo.ab. |
| 56 | drug therapy.fs. |
| 57 | randomly.ab. |
| 58 | trial.ab. |
| 59 | groups.ab. |
| 60 | or/52-59 |
| 61 | 51 or 60 |
| 62 | 43 and 61 |
| 63 | (exp Child/ or Adolescent/ or exp Infant/) not exp Adult/ |
| 64 | 62 not 63 |
| 65 | exp animals/ not humans.sh. |
| 66 | 64 not 65 |
| 67 | limit 66 to yr=2000-2021 |
| 68 | ("new england journal of medicine" or lancet or jama).jn. |
| 69 | 67 and 68 |

Supplementary table 2: Embase search strategy

| 1 | exp heart failure/ |
| --- | --- |
| 2 | ((heart or cardiac* or cardio* or myocardial* or diastolic* or systolic* or paroxysmal*) adj5 (failure* or deem* or deem* or decompensation* or dyspnea* or asthma* or chronic* or insufficient*)).ti,ab,kw. |
| 3 | ((preserved ejection* or reduced ejection*) adj5 fraction*).ti,ab,kw. |
| 4 | (congestive heart* adj5 disease*).ti,ab,kw. |
| 5 | ((cardio renal* or cardiorenal* or reno cardiac* or renocardiac*) adj5 syndrome*).ti,ab,kw. |
| 6 | exp heart arrhythmia/ |
| 7 | (arrhythmia* or dysrhythmia* or bradycardia* or bradyarrhythmia* or tachycardia* or tachyarrhythmia*).ti,ab,kw. |
| 8 | ((irregular* or slow* or rapid* or fast or junctional*) adj3 (heartbeat* or heart beat* or rhythm*)).ti,ab,kw. |
| 9 | ((atrial or auricular or ventricular) adj5 (fibrillation* or flutter*)).ti,ab,kw. |
| 10 | ((heart rhythm* or cardiac rhythm*) adj5 disorder*).ti,ab,kw. |
| 11 | (premature adj3 (atrial or ventricular or junctional or cardiac) adj3 (contraction* or complex*)).ti,ab,kw. |
| 12 | ((accelerat* or junctional*) adj5 rhythm*).ti,ab,kw. |
| 13 | (extra beats or heart block or heart blocks or AV block or AV blocks).ti,ab,kw. |
| 14 | exp cardiac implantable electronic device/ |
| 15 | exp heart pacing/ |
| 16 | exp implantable cardioverter defibrillator/ |
| 17 | exp coronary artery disease/ |
| 18 | (atherosclerosis or atheroscleroses or arteriosclerosis or arterioscleroses or (coronary adj5 disease*)).ti,ab,kw. |
| 19 | (hard* adj3 arter*).ti,ab,kw. |
| 20 | (plaque adj4 build*).ti,ab,kw. |
| 21 | acute coronary syndrome/ |
| 22 | heart infarction/ |
| 23 | percutaneous coronary intervention/ |
| 24 | heart rehabilitation/ |
| 25 | ((cardiac* or cardio* or heart*) adj5 (rehab* or conditioning*)).ti,ab,kw. |
| 26 | exp Aortic Valve/ |
| 27 | exp Aortic Valve Stenosis/ |
| 28 | exp Mitral Valve/ |
| 29 | exp mitral valve stenosis/ |
| 30 | valvular heart disease/ |
| 31 | exp mitral valve regurgitation/ |
| 32 | (left adj2 valv*).af. |
| 33 | (native adj2 valve*).af. |
| 34 | (mitral adj2 valv*).af. |
| 35 | (aortic adj2 valv*).af. |
| 36 | (valve adj2 (disease* or stenos* or insufficien*)).tw. |
| 37 | heart valve replacement/ |
| 38 | heart valve prosthesis/ |
| 39 | (valve adj2 (surg* or replace* or repair* or prosthe* or implant* or procedure*)).tw. |
| 40 | MitraClip.tw. |
| 41 | transcatheter aortic valve implantation/ |
| 42 | TAVI.tw. |
| 43 | or/1-42 [cardiovascular diseases] |
| 44 | exp cohort analysis/ |
| 45 | exp longitudinal study/ |
| 46 | exp prospective study/ |
| 47 | exp follow up/ |
| 48 | cohort$.tw. |
| 49 | exp case control study/ |
| 50 | (case$ and control$).tw. |
| 51 | or/44-50 [BMJ Embase cohort and case-control strategy] |
| 52 | Randomized controlled trial/ |
| 53 | Controlled clinical study/ |
| 54 | random$.ti,ab. |
| 55 | randomization/ |
| 56 | intermethod comparison/ |
| 57 | placebo.ti,ab. |
| 58 | (compare or compared or comparison).ti. |
| 59 | ((evaluated or evaluate or evaluating or assessed or assess) and (compare or compared or comparing or comparison)).ab. |
| 60 | (open adj label).ti,ab. |
| 61 | ((double or single or doubly or singly) adj (blind or blinded or blindly)).ti,ab. |
| 62 | double blind procedure/ |
| 63 | parallel group$1.ti,ab. |
| 64 | (crossover or cross over).ti,ab. |
| 65 | ((assign$ or match or matched or allocation) adj5 (alternate or group$1 or intervention$1 or patient$1 or subject$1 or participant$1)).ti,ab. |
| 66 | (assigned or allocated).ti,ab. |
| 67 | (controlled adj7 (study or design or trial)).ti,ab. |
| 68 | (volunteer or volunteers).ti,ab. |
| 69 | human experiment/ |
| 70 | trial.ti. |
| 71 | or/52-70 |
| 72 | (random$ adj sampl$ adj7 (cross section$ or questionnaire$1 or survey$ or database$1)).ti,ab. not (comparative study/ or controlled study/ or randomi?ed controlled.ti,ab. or randomly assigned.ti,ab.) |
| 73 | Cross-sectional study/ not (randomized controlled trial/ or controlled clinical study/ or controlled study/ or randomi?ed controlled.ti,ab. or control group$1.ti,ab.) |
| 74 | (((case adj control$) and random$) not randomi?ed controlled).ti,ab. |
| 75 | (Systematic review not (trial or study)).ti. |
| 76 | (nonrandom$ not random$).ti,ab. |
| 77 | Random field$.ti,ab. |
| 78 | (random cluster adj3 sampl$).ti,ab. |
| 79 | (review.ab. and review.pt.) not trial.ti. |
| 80 | we searched.ab. and (review.ti. or review.pt.) |
| 81 | update review.ab. |
| 82 | (databases adj4 searched).ab. |
| 83 | (rat or rats or mouse or mice or swine or porcine or murine or sheep or lambs or pigs or piglets or rabbit or rabbits or cat or cats or dog or dogs or cattle or bovine or monkey or monkeys or trout or marmoset$1).ti. and animal experiment/ |
| 84 | Animal experiment/ not (human experiment/ or human/) |
| 85 | or/72-84 |
| 86 | 71 not 85 [Cochrane Embase RCT filter] |
| 87 | 51 or 86 [Cohort or case control studies or Rcts] |
| 88 | 43 and 87 [cardiovascular diseases and cohort or case control studies or RCTs] |
| 89 | (exp adolescent/ or exp child/ or exp infant,newborn/) not exp adult/ |
| 90 | 88 not 89 [adult studies only] |
| 91 | lancet.jn. |
| 92 | new england journal of medicine.jn. |
| 93 | (jama or jama chicago ill or "jama journal of the american medical association").jn. |
| 94 | or/91-93 |
| 95 | 90 and 94 [results limited to specific journals] |
| 96 | limit 95 to yr="2000 - 2021" |
| 97 | remove duplicates from 96 |

Working Group membership: Global Cardiovascular Outcomes Consortium

**Generic**

Nina Ajmone Marsan, Ana G Almeida, Dan Atar, Giuseppe Biondi-Zoccai, Bianca JJM Brundel, Raffaele Bugiardini, Gianluca Campo, Ruben Casado-Arroyo, Claudio Ceconi, Edina Cenko, Michele Ciccarelli, Louise Coats, Gheorghe-Andrei Dan, Victoria Delgado, Polychronis Dilaveris, Dobromir Dobrev, David Duncker, George Giannakoulas, Bruna Gigante, Massimiliano Gnecchi, Jordi Heijman, Bernard Iung, Magnus Jensen, Vijay Kunadian, Malgorzata Lelonek, Erik Lerkevang Grove, Luca Liberale, Riccardo Liga, Roberto Lorusso, Mamas Mamas, Fabio Mangiacapra, María Martín-Fernandez, Jose L Merino, Alessandro Parolari, Cinzia Perrino, Peter P Rainer, Giuseppe MC Rosano, Alexia Rossi, Andrea Rubboli, Tanja Rudolph, Sigrid Sandner, Jolanta Siller-Matula, Samuel Sossalla, Eugenio Stabile, Jurrien ten Berg, Izabella Uchmanowicz, Roland RJ van Kimmenade, Marija Zdravkovic

**ACS-PCI**

Giuseppe Biondi-Zoccai, Marc P Bonaca, Raffaele Bugiardini, Edina Cenko, Massimilano Gnecchi, Erik Lerkevang Grove, Vijay Kunadian, Sergio Leonardi, Luca Liberale, A. Michael Lincoff, Mamas Mamas, Fabio Mangiacapra, Lorenz Räber, Alexia Rossi, Andrea Rubboli, Sigrid Sandner, Jolanta Siller-Matula, Eugenio Stabile, Jurrien ten Berg, Matthias Thielmann

**AF**

Victor Aboyans, Nina Ajmone Marsan, Dan Atar, Bianca JJM Brundel, Ruben Casado-Arroyo, Gheorghe-Andrei Dan, Polychronis Dilaveris, Dobromir Dobrev, Erwan Donal, David Duncker, Erik Lerkevang Grove, Jordi Heijman, Jose L Merino, Cristiano Spadaccio

**HF**

Ana G Almeida, Dan Atar, Antoni Bayés-Genís, Claudio Ceconi, Ovidiu Chioncel, Michele Ciccarelli, Sarah Moharem Elgamal, Justin A Ezekowitz, Gregg Fonarow, George Giannakoulas, Bruna Gigante, Can Gollmann-Tepeköylü, Stephen J Greene, Magnus T Jensen, Malgorzata Lelonek, Luca Liberale, Roberto Lorusso, Marco Metra, Cinzia Perrino, Peter P Rainer, Giuseppe MC Rosano, Gianluigi Savarese, Samuel Sossalla, Roderick W Treskes, Izabella Uchmanowicz, Jacob A Udell, Roland RJ van Kimmenade, Marija Zdravkovic

**TAVI**

Nikolaos Bonaros, Gianluca Campo, Jean-Philippe Collet, Victoria Delgado, Jonathan Howes, Bernard Iung, Vijay Kunadian, Riccardo Liga, Olivia Manfrini, María Martín-Fernandez, Alessandro Parolari, Benyamin Rahmani, Tanja Rudolph, David Tanne

**Data science group clinicians**

Gorav Batra, Asad Bhatty, Chris P Gale, Chris Wilkinson

**Executive committee attendees**

Alan G Fraser, Stefan James, Aldo P Maggioni, Lars Wallentin
